# *De Novo* Characterization of Flower Bud Transcriptomes and the Development of EST-SSR Markers for the Endangered Tree *Tapiscia sinensis*

**DOI:** 10.3390/ijms160612855

**Published:** 2015-06-05

**Authors:** Xiao-Jun Zhou, Yue-Yue Wang, Ya-Nan Xu, Rong-Shan Yan, Peng Zhao, Wen-Zhe Liu

**Affiliations:** 1 School of Life Sciences, Northwest University, 229 Taibai Bei Road, Xi’an 710069, China; E-Mails: plm013@lynu.edu.cn (X.-J.Z.); wyynwu@163.com (Y.-Y.W.); xbdxxuyn@163.com (Y.-N.X.); yanrongshan@126.com (R.-S.Y.); pengzhao@nwu.edu.cn (P.Z.); 2 School of Life Sciences, Luoyang Normal University, 71 Longmen Road, Luoyang 471022, China

**Keywords:** *Tapiscia sinensis*, transcriptome, Illumina sequencing, EST-SSRs (Expressed sequence tag simple sequence repeats)

## Abstract

*Tapiscia sinensis* Oliv (Tapisciaceae) is an endangered species native to China famous for its androdioecious breeding system. However, there is a lack of genomic and transcriptome data on this species. In this study, the *Tapiscia sinensis* transcriptomes from two types of sex flower buds were sequenced. A total of 97,431,176 clean reads were assembled into 52,169 unigenes with an average length of 1116 bp. Through similarity comparison with known protein databases, 36,662 unigenes (70.27%) were annotated. A total of 10,002 (19.17%) unigenes were assigned to 124 pathways using the Kyoto Encyclopedia of Genes and Genomes (KEGG) pathway database. Additionally, 10,371 simple sequence repeats (SSRs) were identified in 8608 unigenes, with 16,317 pairs of primers designed for applications. 150 pairs of primers were chosen for further validation, and the 68 pairs (45.5%) were able to produce clear polymorphic bands. Six polymorphic SSR markers were used to Bayesian clustering analysis of 51 *T. sinensis* individuals. This is the first report to provide transcriptome information and to develop large-scale SSR molecular markers for *T. sinensis*. This study provides a valuable resource for conservation genetics and functional genomics research on *T. sinensis* for future work.

## 1. Introduction

*Tapiscia sinensis* (Tapisciaceae) is a rare and ancient tree species native to China. It is sporadically distributed in the Yangtze River valley and the southwestern provinces of China, as well as in the northern regions of Vietnam [1,2]. *Tapiscia sinensis* is a woody perennial androdioecious species with both male and hermaphrodite individuals in the population [3]. Male and hermaphroditic flowers blossom at similar times and both the male and hermaphroditic flowers produce viable pollen. The flower and fruit grow synchronously on the hermaphroditic individuals of *T. sinensis* [4,5]. Historically, *Tapiscia* was usually placed in Staphyleaceae under Sapindales, and was subsequently classified as Tapisciaceae under Huerteales in APG III; however, its system location has not yet been determined [6,7]. *T. sinensis* is a Quaternary glacial relict plant and could be a good model for inferring the colonization and evolutionary history of post-glacial of plant species in subtropical China [3,8]. In addition, the *T. sinensis* leaves are rich in flavonoid, which have anti-ulcer, antispasmodic, anti-inflammatory and lipid-lowering biological activities, and has, thus, been of great value in medicine [9]. The *T. sinensis* tree is beautiful, with a straight trunk and luxuriant foliage, and it can be used as a garden greening tree species. Therefore, *T. sinensis* is a rare species that has scientific, economic and esthetic value, with excellent germplasm resources. However, due to habitat fragmentation, naturally poor regeneration and the influences of human activity, *T. sinensis* is currently on the International Union for Conservation of Nature (IUCN) red list of threatened species in the vulnerable A1c category [10].

Understanding the genetic relationship and population structure of the *T. sinensis* natural populations is important to develop conservation strategies for this rare and interesting species. However, research on *T. sinensis* at the molecular level remains scarce. At present, only eleven *T. sinensis* simple sequence repeat (SSR) markers have been developed [11]. There is still a serious lack of genomic data on this species, which has seriously hindered developments in the fields of molecular biology and genetics.

A transcriptome includes the full range of mRNA, tRNA, rRNA, and other noncoding RNA molecules expressed by one or a group of cells, organs or tissues in a specific environment or particular developmental stage. Transcriptome sequencing has significant value in molecular marker development, functional gene discovery and differential gene expression [12,13,14]. Since 2005, next-generation sequencing (NGS) technologies, such as Illumina/Solexa, Roche/454 FLX (Flagship Genome Sequencer) and ABI/SOLiD (Supported Oligo Ligation Detetion), have become available [15]. These technologies have proven to be reliable, affordable, and time-saving methods to study non-model organisms’ genomes and transcriptomes. Furthermore, transcriptome sequencing has allowed for the convenient development of additional SSR molecular markers. Compared to traditional methods, the development of SSR markers using transcriptome sequencing techniques is affordable and efficient.

In this study, we used the Illumina sequencing technology to analyze the flower buds transcriptome of *T. sinensis* and to develop a set of simple sequence repeat (SSR) markers. This is the first study attempting to characterize the transcriptome of *Tapiscia.* The transcriptome sequences reported here will provide valuable resources for the development of molecular markers, the molecular genetics research, and novel gene discovery in *T. sinensis*.

## 2. Results and Discussion

### 2.1. Sequencing and de novo Assembly

Next-generation sequencing technology made the transcriptome sequencing lower cost and higher throughput than traditional Sanger sequencing. As one representative of NGS, the Illumina HiSeq™ 2000 sequencing platform has been widely used for transcriptome sequencing in many non-model organisms, including *Liriodendron chinense*, *Cucurbita moschata* and *Houttuynia cordata* [16,17,18].

**Figure 1:**
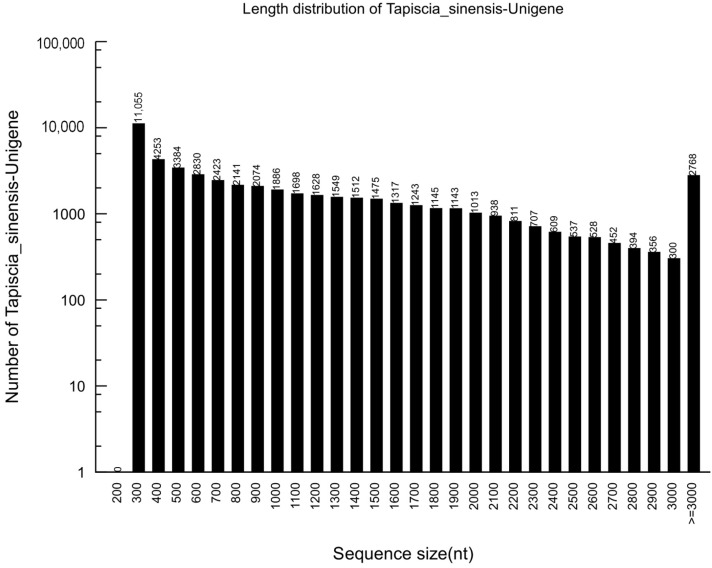
Length distributions of the unigenes in the assembled transcriptome.

In this project, we obtained 97,431,176 clean reads, with a mean length of 100 base pairs (bp). The percentage of Q20, N, and GC was 98.31%, 0.00%, and 45.59%, respectively. The clean reads were *de novo* assembled using Trinity into 55,711 contigs, with a mean length of 1133 bp and an N50 of 1768 bp. The total number of unigenes was 52,169, with a mean length of 1116 bp and an N50 of 1768 bp. Of all of the unigenes, the majority (30,046 unigenes) were in the range of 201 to 1000 bp, which accounted for 57.59%. 13,723 of the unigenes (26.3%) ranged from 1001 to 2000 bp in length, while 8400 of the unigenes (16.1%) had lengths of more than 2000 bp (Figure 1).

The average 1116 bp length of the unigenes was longer than that reported for pumpkin transcriptomes (765 bp) using the Illumina HiSeq™ 2000 sequencing platform [17]. It was also greater than that of the Chinese jujube (473.4 bp) and Siberian apricot (651.6 bp) transcriptomes, using the 454 GS FLX Titanium genomic sequencer platform [19,20].

### 2.2. Sequence Annotation and Classification

**Figure 2:**
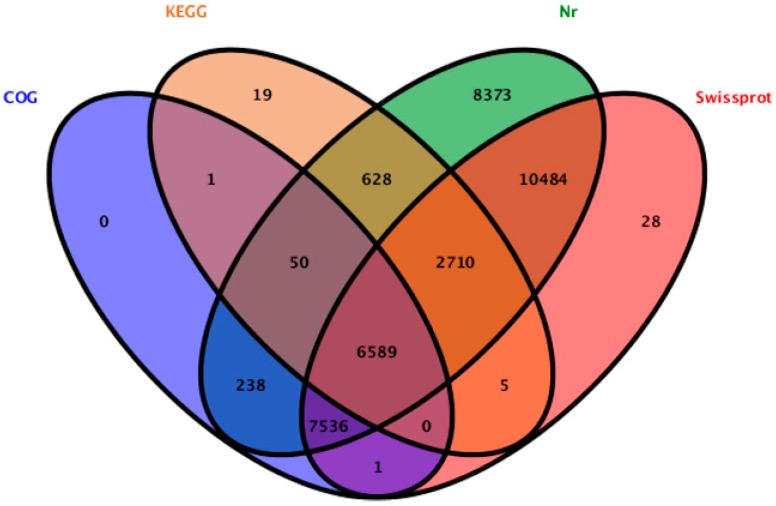
The unigenes showed homology with sequences in the Nr, Swiss-Prot, COG (Clusters of Orthologous Groups of proteins) and KEGG (Kyoto Encyclopedia of Genes and Genomes) databases.

We searched the 52,169 unigenes against the protein databases. 36,662 unigenes (70.27%) were annotated, 15,507 unigenes cannot be aligned to any database remained without annotation. In the annotated unigenes, 36,608 (70.17%), 27,353 (52.43%), 14,415 (27.63%) and 10,002 (19.17%) unigenes showed homology with sequences in the Nr (Non redundant), Swiss-Prot, COG (Clusters of Orthologous Groups of proteins), and KEGG databases, respectively (Figure 2). In the annotated unigenes in the Nr database, most samples (13,776 unigenes) matched the “theobroma cacao” proteins, followed by “vitis vinifera” (8474 unigenes), “fragaria vesca subsp. Vesca” (2058 unigenes), “cucumis sativus” (1887 unigenes) and “oryza sativa Japonica Group” (1317 unigenes). In the 15,507 un-annotated unigenes, 835 unigenes were established nucleotide sequence direction (5′-3′) and amino sequence of the predicted coding region by using ESTScan [21].

**Figure 3:**
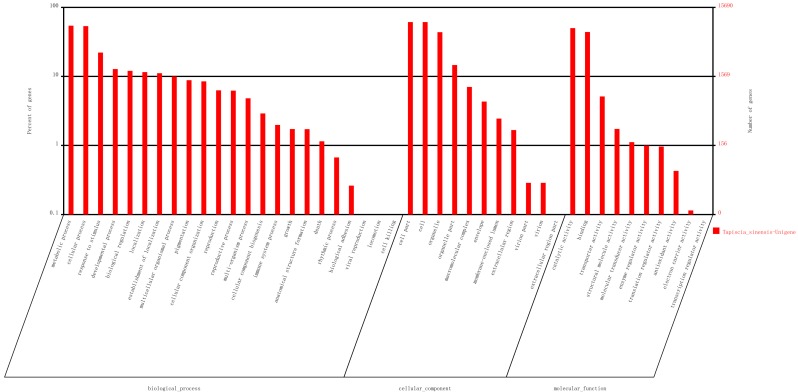
Unigenes were assigned to Gene Ontology.

Based on Nr annotation, 15,690 unigenes were assigned gene ontology (GO) with 83,842 functional terms. In total, 36,497 terms were assigned to the biological processes group, 30,925 terms were assigned to the cellular components group and 16,420 terms were assigned to the molecular functions group (Figure 3). Of the biological process category, metabolic process (8526, 23.36%) and cellular process (8406, 23.03%) were the most frequent terms used. Genes related to response to stimulus (3476 unigenes), anatomical structure formation (270 unigenes), and rhythmic process (105 unigenes) were also identified in this group. In the class of “response to stimulus”, unigene0008775, unigene0005239, and unigene0049385, that were respectively annotated as “Pedicel, carpel, stamen, petal differentiation and expansion stage, group 2-like protein”, “Phytochrome and flowering time regulatory protein isoform 2”, and “Protein early flowering 3”, which putatively related to response to environmental stimulus were discovered. In the class of “rhythmic process”, unigene0044321 and unigene0017817, which were respectively annotated as “circadian clock-associated FKF1” and “Protein EARLY FLOWERING 4 OS”, were found. In the class of “anatomical structure formation”, unigene0011847 was annotated as “Sex determination protein tasselseed-2-like” was found. These unigenes may involve in flower development, differentiation and flowering time determination in *T. sinensis*. Regarding the cellular components category, the cell and cell parts were the most prominent terms. In the molecular functions category, catalytic activity (7861 unigenes) and binding (6905 unigenes) were the most highly represented terms.

**Figure 4:**
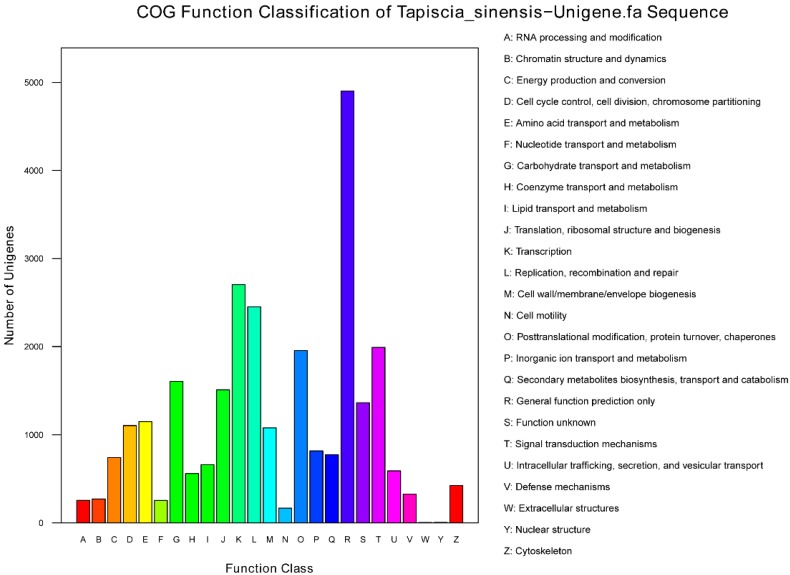
Unigenes were assigned to COG classifications.

Furthermore, all unigenes were subjected to a search against the Clusters of Orthologous Groups of proteins (COG) database for functional prediction and classification, and 27,672 sequences were assigned to COG classifications (Figure 4). Of the 25 COG categories, the cluster for “general function prediction only” (4902, 17.71%) was the largest group, followed by “transcription” (2704, 9.77%), “replication, recombination and repair” (2451, 8.86%), “signal transduction mechanisms” (1991, 7.19%) and “posttranslational modification, protein turnover, chaperones” (1955, 7.06%). Additionally, 1605 unigenes were assigned to “carbohydrate transport and metabolism” group, which include unigene0027247 that was annotated as “Glycogen synthase”, unigene0027034 annotated as “6-phosphofructokinase”, and unigene0026072 annotated as “Glyceraldehyde-3-phosphate dehydrogenase/erythrose-4-phosphate dehydrogenase”; 743 unigenes were assigned to “energy production and conversion”, which include unigene0032350 annotated as “Fumarase”, unigene0032531 annotated as “Glycerol-3-phosphate dehydrogenase”, unigene0038751 annotated as “Nicotinamide-adenine dinucleotid dehydrogenase, Flavin adenine dinucleotide containing subunit”, and unigene0039174 annotated as “Phosphoenolpyruvate carboxykinase (ATP)”. These key genes laid the foundation for the study of *T. sinensis*.

To identify the biological pathways in *T. sinensis*, the annotated unigenes were mapped to the reference of typical pathways in the KEGG database. In this study, 10,002 (19.17%) unigenes were assigned to 124 pathways, which include pathways about “Starch and sucrose metabolism” (224 unigenes), “Terpenoid backbone biosynthesis” (72 unigenes), and “Carbon fixation in photosynthetic organisms” (136 unigenes). The top five pathways in KEGG analysis consist of “Metabolic pathways” (ko01100), “Biosynthesis of secondary metabolites” (ko01110), “Ribosomes” (ko03010), “Spliceosome” (ko03040), and “RNA transport” (ko03013) (Table S1). It is worth noting that 260 (2.6%) unigenes in the ‘‘Plant hormone signal transduction’’ pathway were found in the pathways of auxin, cytokinine, gibberellins, abscisic acid, ethylene, brassinosteroid, jasmonic acid and salicylic acid, they may be involved in the regulation of “Cell enlargement”, “Plant growth”, “Shoot initiation”, and “Senescence” in *T. sinensis* flowers (Figure S1).

### 2.3. Genes Putatively Related to Flower Development

**Figure 5:**
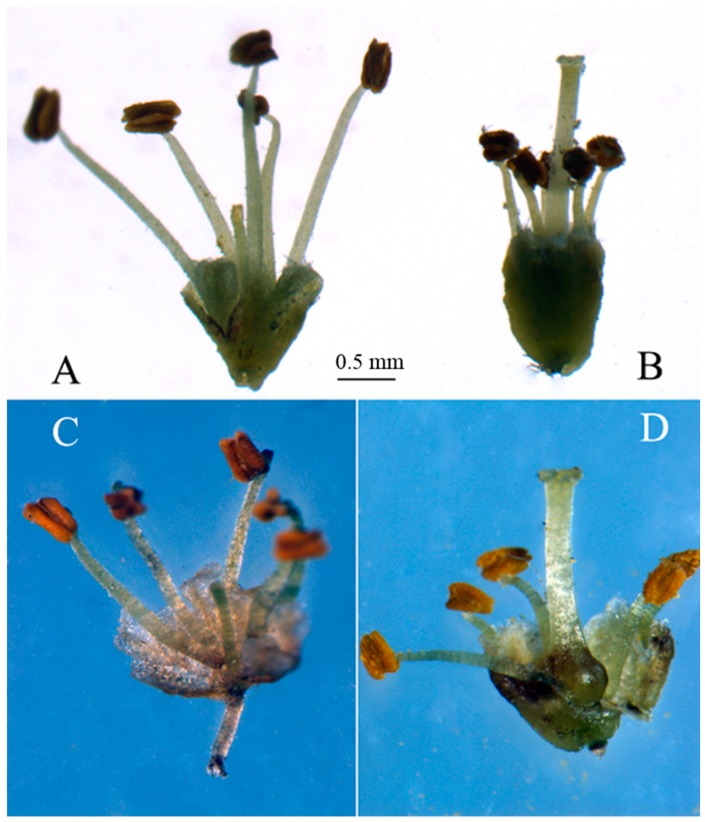
The male flower and hermaphrodite flower of *T. sinensis*, (**A**) and (**C**) are flowers from male individual; (**B**) and (**D**) are bisexual flowers from hermaphrodite individual. Anatomical images (**C**,**D**) are the difference between male and hermaphrodite flowers about pistils. Picture (**C**) shows degenerated pistil, picture (**D**) shows well-developed pistil.

In the natural population of *T. sinensis*, male and hermaphrodite individuals coexist. The flowers of male individuals have normal stamens and degenerated pistil, and the hermaphrodite flowers have normal pistil and stamens (Figure 5). In this study we identified several unigenes that are putative homologs of *Arabidopsis thaliana*, *Populus tomentosa*, *Theobroma cacao* and *Vitis vinifera* genes that are involved in floral development. These include genes that flowering time determination, such as the MADS box genes *SOC1* and *Flowering Locus C*, and perceive or respond to environmental signals, such as *FKF1* and *ELF4*, and Sex determination protein *tasselseed-2* (Table S2)*.*

Flowering is an important developmental process for the reproduction of the plant. It is regulated by environmental and genetic factors. The MADS box genes *SOC1* and *Flowering Locus C* (*FLC*) of the plant *Arabidopsis thaliana* have been shown to have an important role in the integration of molecular flowering time pathways [22,23]. These genes are essential for the correct timing of flowering, and help to ensure that fertilization occurs at the time of maximal reproductive potential. The transition of vegetative to flowering in plants is coordinated by external cues such as day length (photoperiod) or temperature. Day-length sensing involves an interaction between the relative length of day and night, and endogenous rhythms that are controlled by the plant circadian clock. The genes “*EARLY FLOWERING 4 (ELF4)*” and “*FLAVIN-BINDING, KELCH REPEAT, F-BOX (FKF1)*” are involved in circadian regulation and photoperiod perception [24,25]. The unigenes were annotated as sex determination protein tasselseed-2 (Ts-2) has been shown to be involved in the arrest of developing pistils in male flowers of the grass family (Poaceae), *Zea mays* and *Tripsacum dactyloides* [26,27]. Maize through programmed abortion of preformed organ primordia produces separate unisexual flowers.

By comparing the unigenes that first assembled separately in male and hermaphrodite samples, we found that the expression of the above-mentioned unigenes had no significant difference in the two types of flowers, except unigene0052119 and unigene0050634. The unigene0052119 was annotated as “ELF4-like protein” over expression in flowers of hermaphrodites, which may involve in circadian regulation and photoperiod perception in *T. sinensis*. The unigene0050634 was annotated as “sex determination protein tasselseed-2-like” over expression in flowers of males, which may involve in the arrest of developing pistils in male flowers. These unigenes are important resources for the study of floral development and flower organ formation of *T. sinensis* in the future.

### 2.4. SSR Mining from the T. sinensis Transcriptome

SSRs are useful as molecular markers for genetics and biology researches. All 52,169 unigenes generated from the transcriptome were used to develop potential SSR loci using MISA (MIcroSAtellite identification) software. A total of 10,371 SSRs were identified in 8608 unigenes, with 5439 sequences suit to designed primers, and 16,317 pairs of primers designed for application. Of all of the SSR containing sequences, 1436 sequences contained more than one SSR, and 733 SSRs were present in compound forms (Table 1).

**Table 1:** Summary of the EST-SSRs identified in the transcriptome sequences.

Item	Number
Total number of sequences examined	52,169
Total size of examined sequences (bp)	58,253,075
Total number of identified SSRs	10,371
Number of SSR containing sequences	8608
Number of sequences containing more than 1 SSR	1436
Number of SSRs present in compound formation	733

**Figure 6:**
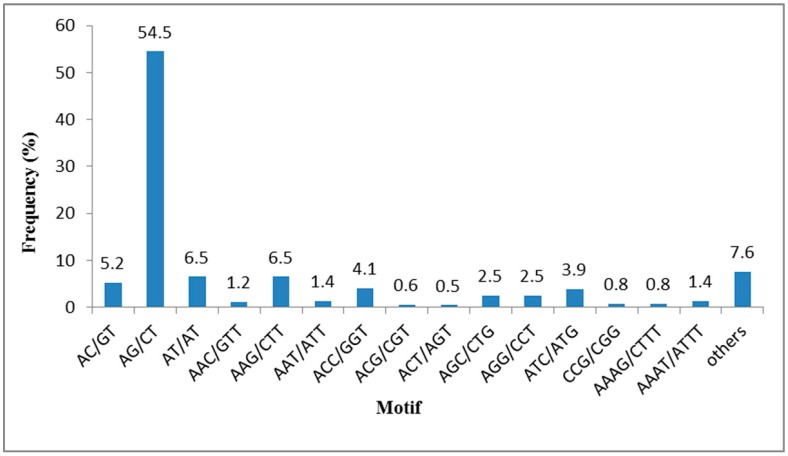
Frequency distribution of SSRs based on the motif types.

Of the 10,371 identified SSRs, the most abundant repeat motif types were di-nucleotide repeat motifs (6888, 66.42%), followed by tri-nucleotide (2486, 23.97%), tetra-nucleotide (522, 5.03%), hexa-nucleotide (296, 2.85%) and penta-nucleotide (179, 1.73%) repeat motifs. The frequencies of the SSRs with different numbers of tandem repeats are summarized in Table 2. SSRs with six tandem repeats were the most common (2797, 26.97%), followed by seven tandem repeats (1831, 17.66%), five tandem repeats (1447, 13.95%), and nine tandem repeats (1256, 12.11%). The most common type of SSR motif was AG/CT which accounted for 54.5%, followed by AT/AT and AAG/CTT (6.5%), AC/GT (5.2%), ACC/GGT (4.1%) (Figure 6). This result is consistent with previous research showing di-nucleotide repeats to be the most abundant type, followed by were tri-nucleotide repeats [17,28,29]. The most abundant di-nucleotide motifs AG/CT and tri-nucleotide motifs AAG/CTT were also coincident with earlier research [17].

In the 8608 SSR-containing ESTs, the most of them are functional. These ESTs are useful for the SSR identification of related to flower types and some other important phenotypes, such as stress resistance. The detail and functional description of 8608 SSR-containing EST accessions were shown in Tables S3 and S4.

**Table 2:** The distribution of the identified EST-SSRs in sequences using the MISA (MIcroSAtellite identification) software.

Repeat Numbers	Motif					Total	Repeat Number (%)
	Di-	Tri-	Tetra-	Penta-	Hexa		
4	0	0	368	158	266	792	7.64
5	0	1319	101	15	12	1447	13.95
6	2026	703	52	5	11	2797	26.97
7	1416	411	0	0	4	1831	17.66
8	1164	49	1	1	2	1217	11.73
9	1256	0	0	0	0	1256	12.11
10	823	1	0	0	0	824	7.95
11	195	1	0	0	0	196	1.89
˃11	8	2	0	0	1	11	0.11
Total	6888	2486	522	179	296	10,371	100
Motif (%)	66.42	23.97	5.03	1.73	2.85		

### 2.5. SSR Marker Assessment and Application

**Figure 7:**
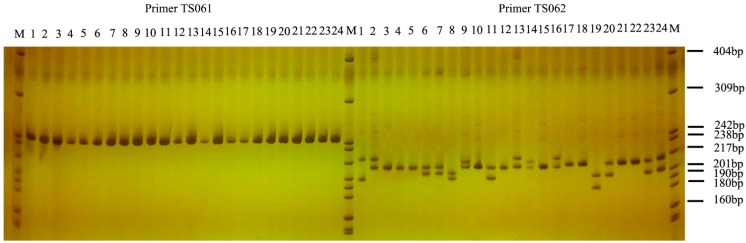
The results of the SSR-PCR that produced clear bands. M refers to DNA markers pBR322 DNA/MspI. Numbers 1 to 24 represent twenty-four individuals of *T. sinensis.* The products of primer TS061 are single bands without polymorphism, while those of TS062 are clear polymorphic bands.

A suit of 150 pairs of primers was synthetized and assessed in the 24 *T. sinensis* individuals. The result shows that, 117 pairs (78%) successfully amplified target fragments for SSR-PCR (Simple Sequence Repeat Polymerase Chain Reaction), including 68 pairs that were able to produce clear polymorphic bands (45.5%) and 49 pairs of primers that were able to produce single bands without polymorphism. The results of the SSR-PCR amplification are shown as examples in Figure 7. Of 68 polymorphic SSR markers (*N* = 24), the detected number of alleles per locus ranged from 2 to 6 with an average of 3.24 alleles per locus, and the observed and expected heterozygosity varied from 0.000 to 1.000 and from 0.042 to 0.782, respectively. Polymorphic information content varied 0.040 to 0.729. Details of the 68 polymorphic SSR markers from *T. sinensis* are shown in Table S5. In addition, the Bayesian clustering analysis of the 51 individuals was constructed based on six polymorphic SSR markers, with the characteristics shown in Table 3. The analysis indicated that we developed SSR markers can differentiate population genetic differences of different geographic locations, dividing the 51 individuals into two main groups of 29 (fromWuling Mountains) and 22 (from Qinling Mountains) (Figure S2).

In recent years, EST-SSR has been widely used in the studies of plant at the DNA level. In 2014, Guo *et al.* used forty effective EST-SSR to test the genetic diversity of 15 hexaploid oat germplasm resources, and clustering them into three groups according to species. The forty primers showed higher availability on the study of 31 chromosome ploidy unknown oat germplasm resources, and some diploids and tetraploids of oat germplasm resources were found in them [30]. Wang *et al.* used 28 EST-SSR derived from alfalfa and soybean to study the genetic relationship of 26 accessions representing four species of *Crotalaria*, and re determined the phylogenetic classification of the 26 accessions [31]. Recently, nineteen EST-SSR analyses provide insights about genetic relatedness, population structure and gene flow in grass pea of 176 accessions [32]. In this study, we developed EST-SSR could be used for genetic diversity analysis, identification of Germplasm Resources and the molecular phylogeny and evolution in Tapisciaceae.

**Table 3:** Characteristics of the six primer pairs used for the phylogenetic analysis of the 51 individuals.

Primer	Motif	*K*	*H_O_*	*H_E_*	*PIC*	HW
TS053	(TCA)6	9	0.706	0.835	0.804	ND
TS126	(TAC)7	6	0.706	0.768	0.723	NS
TS103	(AAGA)5	5	0.549	0.631	0.573	NS
TS149	(TTG)11	6	0.608	0.769	0.725	NS
TS060	(TGT)6	6	0.529	0.501	0.464	NS
TS013	(AGA)8	5	0.51	0.653	0.592	NS

*K*, No. (Number) of alleles; *H_O_*, Observed heterozygosity; *H_E_*, Expected heterozygosity; *PIC*, Polymorphism Information Content; HW, Hardy-Weinberg equilibrium test; NS, Not significant; ND, Not done.

For comparison with existing databases, functional annotation and SSR identification using PCR amplification, the results indicated that the quality of the *T. sinensis* transcriptome sequencing and assembly was very high. To date, this is the first research on *T. sinensis* transcriptome sequencing and analysis. These data will lay the foundations for the future research on functional gene identification, molecular genetics, physiology and biochemistry of *T. sinensis*.

## 3. Materials and Methods

### 3.1. Plant Material and RNA Extraction

The inflorescences of three male and three hermaphrodite individuals were collected separately at Northwest University, Shanxi, China (34°15′N, 108°55′E, H 400 m) in April 2014. The samples were between 0.2–1.0 cm long and inserted a lot of small flower buds. The total RNA of above listed six samples were isolated separately using the Trizol Kit (Promega, Beijing, China) following by the manufacturer’s instructions, then were treated with RNase-free DNase I (Takara Biotech Incorporation, Otsu, Japan) for 30 min at 37 °C to remove residual DNA. RNA quality was verified using a 2100 Bioanalyzer (Agilent Technologies, Santa Clara, CA, USA) and were also checked by RNase free agarose gel electrophoresis. The equal amounts of RNA from the three same sex individuals were mixed together. Then, the two total RNA samples, one from three male individuals and another from three hermaphroditic individuals, were prepared to following experiment.

The subsequent steps, which include two cDNA libraries construction, Illumina sequencing, and first assembled of the two samples were carried out separately.

### 3.2. cDNA Library Construction and Illumina Sequencing

In brief, Poly (A) mRNA was enriched by Oligo (dT) magnetic beads. All mRNA was cut into short fragments by adding fragmentation buffer. Taking these short fragments as templates, random hexamer primers were used to synthesize first-strand cDNA. Second-strand cDNA was synthesized using the first-strand cDNA as templates in the presence of buffer, dNTPs, RNase H and DNA polymerase I. The short fragments were purified using the QIAquick PCR extraction kit (Qiagen, Shanghai, China) and resolved with EB buffer for end reparation and A tailing. The short fragments were then connected with sequencing adapters. Following agarose gel electrophoresis and extraction of cDNA from gels, the cDNA fragments were purified and enriched by PCR to construct the final cDNA library for sequencing. Illumina sequencing was performed at Gene Denovo Co. Guangzhou, China using the HiSeq™ 2000 platform. The sequencing data is available from the the National Center for Biotechnology Information (NCBI) Short Read Archive (SRA, Bethesda, Maryland, MD, USA. Accession numbers: PRJNA284864).

### 3.3. De Novo Assembly and Annotation

The raw sequencing reads were first filtered by removing invalid reads, including reads with adaptor contamination, with ambiguous “N” bases at a ratio greater than 5% and reads with more than 50% base with quality lower than 20 in one sequence. The short clean reads were assembled using Trinity software to construct contigs [33]. These contigs were used for the further process of sequence clustering with the software TGICL (TIGR Gene Indices clustering tools) to form unigenes [34]. The reads from the male flower buds and hermaphrodite flower buds were first assembled separately, then assembled together to acquire non-redundant unigenes that were as long as possible.

All of the unigenes were aligned to sequences in the four protein databases, such as Nr (NCBI non-redundant database), Swiss-Prot, COG and KEGG (the Kyoto Encyclopedia of Genes and Genomes), using blastx with an E-value threshold <1 × 10^−5^. The sequence direction of the unigenes was determined according to the best alignment results. When the results were conflicted among databases, the direction was determined consecutively by Nr, Swiss-Prot, KEGG and COG. When a unigene would not align to any database, ESTScan was used to predict coding regions and determine sequence direction [21]. GO annotations for the unigenes were achieved using the Blast2GO software (Valencia, Spain) [35]. Functional classification of the unigenes was performed using WEGO (web tool for plotting GO annotations) software (Hangzhou, China) [36].

### 3.4. SSR Detection and Primer Design

The 52,169 unigenes were searched for Simple Sequence Repeats (SSRs) using the MISA program (MIcroSAtellite identification tool). The parameters were based on the minimum number of repeats, which were set as follows: six repeat units for di-nucleotides, five for tri-nucleotides, and four for tetra-, penta- and hexa-nucelotides. The maximum interruption distance between two SSRs was specified as 100 bases. Primer pairs of each SSR loci were designed using the Primer3 program in the flanking regions of SSRs [37]. Primers were designed based on the following criteria: (1) GC content ranged between 40% and 60%; (2) Primer length between 18 and 27 bp; (3) Melting temperature between 55 and 63 °C; and (4) The expected PCR product sizes ranged from 100 to 280 bp.

### 3.5. SSR Validation and Assessment

To validate the EST-SSR markers developed from the transcriptome, fifty-one *T. sinensis* adult individuals, of which 22 from the Qinling Mountains (QL) (33°31′N, 108°35′E, H 1348 m) and 29 from the Wuling Mountains (WL) of China (28°47′N, 110°15′E, H 490 m), were collected.

A total of 150 primer pairs were synthesized and twenty-four of the fifty-one samples were selected randomly to validate the polymorphism of the SSR markers. After detecting Hardy-Weinberg equilibrium and linkage disequilibrium between microsatellites by using Cervus 3.0 and Genepop [38,39], six polymorphic SSR markers were selected for Bayesian clustering analysis on the 51 *T. sinensis* individuals using Structure v2.3 software (Stanford, CA, USA). [40]. The analysis was ran with the parameters as: 30,000 Burn-in Period, 100,000 MCMC Reps (Markov Chain Monte Carlo Repetitions), three runs were conducted for each *K* (1–8). STRUCTURE HARVESTER were used to calculate ΔK values and identified the suitable value of *K* = 2 for the population structure [41].

## 4. Conclusions

In this study, the transcriptome of *T. sinensis* was analyzed using high-throughput Illumina sequencing. Through *de novo* assembly and sequence annotation, we obtained 52,169 sequences and identified some important characteristics of the *T. sinensis* transcriptome. In addition, we identified and characterized 10,371 potential SSR markers. A set of 150 SSR markers were selected to detect polymorphisms among the 24 *T. sinensis* individuals. A total of 117 pairs (78%) had successfully amplified target fragments, with 68 pairs showing polymorphic loci. This study is the first application of Illumina sequencing technology used to investigate the transcriptome and assemble RNA-seq reads without a reference genome for *T. sinensis*. The SSRs and sequences will provide useful resources for conservation genetics and functional genomics research on *T. sinensis* in the future.

## Supplementary Files

Supplementary File 1
