# Supplementary material for: De Novo Characterization of Flower Bud Transcriptomes and the Development of EST-SSR Markers for the Endangered Tree Tapiscia sinensis
Source: Int J Mol Sci. 2015 Jun 5;16(6):12855–70. doi: 10.3390/ijms160612855 (PMC4490475; doi:10.3390/ijms160612855)
Supplement: Supplementary File 1 [file ijms-16-12855-s001.zip › ijms-84175-Supplementary Information/ijms-84175-Supplementary Information.pdf]

Supplementary Information

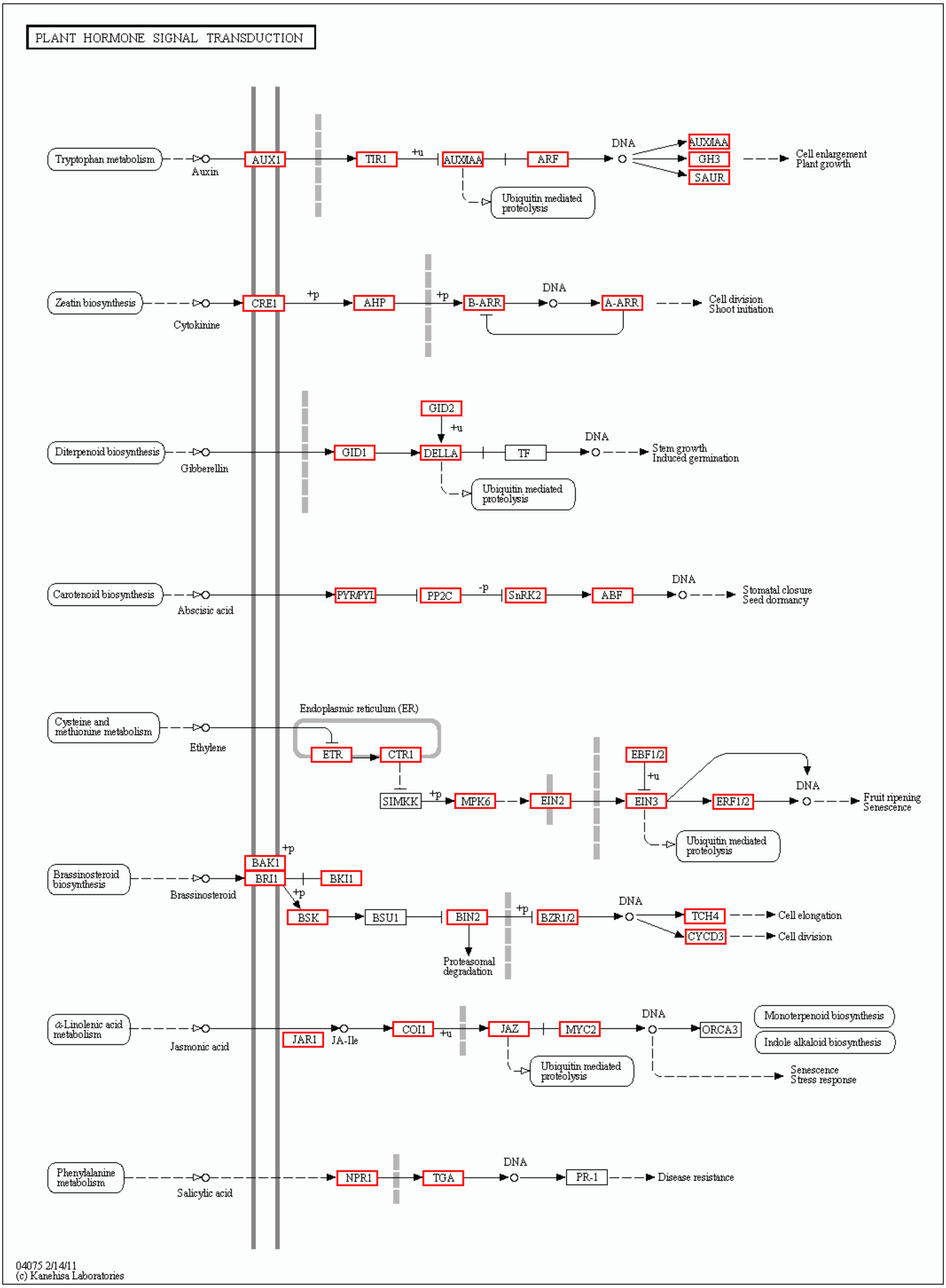

Figure S1. Unigenes were in the "Plant hormone signal transduction" pathways.

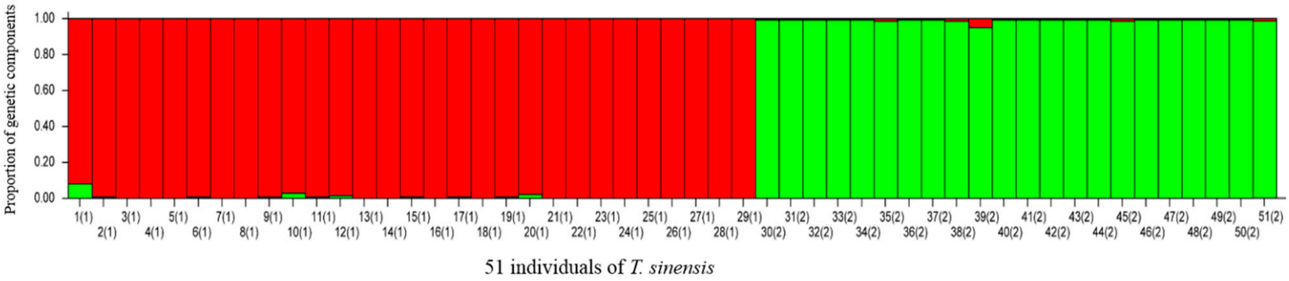

Figure S2. Bayesian clustering analysis of the 51 individuals was constructed based on the six polymorphic SSR markers. Red means genetic cluster in Wuling Mountains; Green means genetic cluster in Qinling Mountains. Individuals 1 to 29 were fromWuling Mountains while individuals 30 to 51 were from Qinling Mountains.

Table S1. Unigenes were assigned to 124 pathways.

Number	Pathway	All Genes with Pathway Annotation (10002)	Pathway ID
1	Metabolic pathways	2587 (25.86%)	ko01100
2	Biosynthesis of secondary metabolites	1263 (12.63%)	ko01110
3	Ribosome	337 (3.37%)	ko03010
4	Spliceosome	313 (3.13%)	ko03040
5	RNA transport	273 (2.73%)	ko03013
6	Protein processing in endoplasmic reticulum	263 (2.63%)	ko04141
7	Plant hormone signal transduction	260 (2.6%)	ko04075
8	Purine metabolism	259 (2.59%)	ko00230
9	Oxidative phosphorylation	233 (2.33%)	ko00190
10	Starch and sucrose metabolism	224 (2.24%)	ko00500
11	Plant-pathogen interaction	217 (2.17%)	ko04626
12	Ribosome biogenesis in eukaryotes	210 (2.1%)	ko03008
13	Ubiquitin mediated proteolysis	207 (2.07%)	ko04120
14	RNA degradation	199 (1.99%)	ko03018
15	mRNA surveillance pathway	196 (1.96%)	ko03015
16	Pyrimidine metabolism	191 (1.91%)	ko00240
17	Glycolysis/Gluconeogenesis	180 (1.8%)	ko00010
18	Amino sugar and nucleotide sugar metabolism	168 (1.68%)	ko00520
19	Glycerophospholipid metabolism	139 (1.39%)	ko00564
20	Carbon fixation in photosynthetic organisms	136 (1.36%)	ko00710
21	Endocytosis	135 (1.35%)	ko04144
22	Peroxisome	133 (1.33%)	ko04146
23	Phagosome	128 (1.28%)	ko04145
24	Glycine, serine and threonine metabolism	122 (1.22%)	ko00260
25	Cysteine and methionine metabolism	118 (1.18%)	ko00270
26	Pyruvate metabolism	115 (1.15%)	ko00620

Table S1. *Cont.*

Number	Pathway	All Genes with Pathway Annotation (10002)	Pathway ID
27	Aminoacyl-tRNA biosynthesis	109 (1.09%)	ko00970
28	Nucleotide excision repair	108 (1.08%)	ko03420
29	Arginine and proline metabolism	104 (1.04%)	ko00330
30	Phenylpropanoid biosynthesis	102 (1.02%)	ko00940
31	Glutathione metabolism	102 (1.02%)	ko00480
32	Homologous recombination	95 (0.95%)	ko03440
33	Glyoxylate and dicarboxylate metabolism	94 (0.94%)	ko00630
34	Basal transcription factors	90 (0.9%)	ko03022
35	Pentose phosphate pathway	89 (0.89%)	ko00030
36	Inositol phosphate metabolism	87 (0.87%)	ko00562
37	Fructose and mannose metabolism	87 (0.87%)	ko00051
38	Porphyrin and chlorophyll metabolism	83 (0.83%)	ko00860
39	Photosynthesis	83 (0.83%)	ko00195
40	Glycerolipid metabolism	81 (0.81%)	ko00561
41	*N*-Glycan biosynthesis	80 (0.8%)	ko00510
42	Base excision repair	79 (0.79%)	ko03410
43	Protein export	78 (0.78%)	ko03060
44	Phosphatidylinositol signaling system	78 (0.78%)	ko04070
45	Pentose and glucuronate interconversions	78 (0.78%)	ko00040
46	RNA polymerase	76 (0.76%)	ko03020
47	Valine, leucine and isoleucine degradation	75 (0.75%)	ko00280
48	DNA replication	74 (0.74%)	ko03030
49	Terpenoid backbone biosynthesis	72 (0.72%)	ko00900
50	Galactose metabolism	72 (0.72%)	ko00052
51	Alanine, aspartate and glutamate metabolism	71 (0.71%)	ko00250
52	Citrate cycle (TCA cycle)	71 (0.71%)	ko00020
53	Mismatch repair	69 (0.69%)	ko03430
54	Proteasome	69 (0.69%)	ko03050
55	Phenylalanine, tyrosine and tryptophan biosynthesis	68 (0.68%)	ko00400
56	Valine, leucine and isoleucine biosynthesis	68 (0.68%)	ko00290
57	Nitrogen metabolism	67 (0.67%)	ko00910
58	Phenylalanine metabolism	67 (0.67%)	ko00360
59	Fatty acid metabolism	64 (0.64%)	ko00071
60	Ubiquinone and other terpenoid-quinone biosynthesis	63 (0.63%)	ko00130
61	alpha-Linolenic acid metabolism	59 (0.59%)	ko00592
62	Ascorbate and aldarate metabolism	57 (0.57%)	ko00053

Table S1. *Cont.*

Number	Pathway	All Genes with Pathway Annotation (10002)	Pathway ID
63	SNARE interactions in vesicular transport	57 (0.57%)	ko04130
64	Pantothenate and CoA biosynthesis	55 (0.55%)	ko00770
65	Cyanoamino acid metabolism	54 (0.54%)	ko00460
66	Glycosylphosphatidylinositol-anchor biosynthesis	53 (0.53%)	ko00563
67	β-Alanine metabolism	53 (0.53%)	ko00410
68	Tyrosine metabolism	53 (0.53%)	ko00350
69	Fatty acid biosynthesis	51 (0.51%)	ko00061
70	Ether lipid metabolism	50 (0.5%)	ko00565
71	Natural killer cell mediated cytotoxicity	49 (0.49%)	ko04650
72	Biosynthesis of unsaturated fatty acids	49 (0.49%)	ko01040
73	Propanoate metabolism	48 (0.48%)	ko00640
74	Carotenoid biosynthesis	46 (0.46%)	ko00906
75	Sphingolipid metabolism	45 (0.45%)	ko00600
76	Sulfur metabolism	44 (0.44%)	ko00920
77	One carbon pool by folate	43 (0.43%)	ko00670
78	Circadian rhythm-plant	43 (0.43%)	ko04712
79	Photosynthesis-antenna proteins	41 (0.41%)	ko00196
80	Histidine metabolism	38 (0.38%)	ko00340
81	Folate biosynthesis	36 (0.36%)	ko00790
82	Regulation of autophagy	35 (0.35%)	ko04140
83	Selenocompound metabolism	34 (0.34%)	ko00450
84	Diterpenoid biosynthesis	32 (0.32%)	ko00904
85	Steroid biosynthesis	30 (0.3%)	ko00100
86	Flavonoid biosynthesis	29 (0.29%)	ko00941
87	Linoleic acid metabolism	29 (0.29%)	ko00591
88	Tropane, piperidine and pyridine alkaloid biosynthesis	29 (0.29%)	ko00960
89	Limonene and pinene degradation	27 (0.27%)	ko00903
90	Fatty acid elongation in mitochondria	27 (0.27%)	ko00062
91	Isoquinoline alkaloid biosynthesis	26 (0.26%)	ko00950
92	Tryptophan metabolism	26 (0.26%)	ko00380
93	Nicotinate and nicotinamide metabolism	26 (0.26%)	ko00760
94	Glycosphingolipid biosynthesis-globo series	25 (0.25%)	ko00603
95	Lysine degradation	24 (0.24%)	ko00310
96	Stilbenoid, diarylheptanoid and gingerol biosynthesis	23 (0.23%)	ko00945
97	Lysine biosynthesis	23 (0.23%)	ko00300
98	Butanoate metabolism	22 (0.22%)	ko00650
99	Glycosaminoglycan degradation	21 (0.21%)	ko00531
100	Other glycan degradation	21 (0.21%)	ko00511
101	Sulfur relay system	21 (0.21%)	ko04122

Table S1. *Cont.*

Number	Pathway	All Genes with Pathway Annotation (10002)	Pathway ID
102	Zeatin biosynthesis	20 (0.2%)	ko00908
103	Thiamine metabolism	19 (0.19%)	ko00730
104	Riboflavin metabolism	19 (0.19%)	ko00740
105	Circadian rhythm-mammal	18 (0.18%)	ko04710
106	Flavone and flavonol biosynthesis	17 (0.17%)	ko00944
107	Non-homologous end-joining	15 (0.15%)	ko03450
108	Arachidonic acid metabolism	14 (0.14%)	ko00590
109	Monoterpenoid biosynthesis	14 (0.14%)	ko00902
110	Glucosinolate biosynthesis	13 (0.13%)	ko00966
111	Brassinosteroid biosynthesis	13 (0.13%)	ko00905
112	Vitamin B6 metabolism	12 (0.12%)	ko00750
113	Taurine and hypotaurine metabolism	10 (0.1%)	ko00430
114	C5-Branched dibasic acid metabolism	9 (0.09%)	ko00660
115	Lipoic acid metabolism	8 (0.08%)	ko00785
116	Synthesis and degradation of ketone bodies	7 (0.07%)	ko00072
117	Glycosphingolipid biosynthesis-ganglio series	6 (0.06%)	ko00604
118	Sesquiterpenoid biosynthesis	6 (0.06%)	ko00909
119	Other types of *O*-glycan biosynthesis	4 (0.04%)	ko00514
120	Caffeine metabolism	4 (0.04%)	ko00232
121	ABC transporters	3 (0.03%)	ko02010
122	Biotin metabolism	3 (0.03%)	ko00780
123	Indole alkaloid biosynthesis	2 (0.02%)	ko00901
124	Anthocyanin biosynthesis	1 (0.01%)	ko00942

Table S2. *T. sinense* unigenes that share homology with flower develop genes.

Gene ID	Annotation	Annotation Databases	*E*-Value
Unigene0044321	Circadian clock-associated Flavin-biding, kelch repeat, F-box (FKF1) [*Glycine max*]	Nr (Non redundant)	0
Unigene0015678	Early flowering -like 4 [*Theobroma cacao*]	Nr	2.00×10^{-53}
Unigene0017570	Flowering locus C [*Betula platyphylla*]	Nr	2.00×10^{-14}
Unigene0017571	Flowering locus C [*Vitis vinifera*]	Nr	2.00×10^{-23}
Unigene0010567	Flowering locus T protein [*Betula platyphylla*]	Nr	3.00×10^{-43}
Unigene0030535	Flowering time control protein abscisic acid receptor (FCA) Organism species (OS) = *Arabidopsis thaliana* Gene name(GN) = FCA Protein Existence (PE) = 1 SequenceVersion (SV) = 2	Swissprot	5.00×10^{-24}
Unigene0023453	Flowering time control protein autonomous-pathway component (FPA) OS = *Arabidopsis thaliana* GN = FPA PE = 2 SV = 2	Swissprot	2.00×10^{-121}
Unigene0015676	Flowering-promoting factor 1-like protein 1 Organism species (OS) = *Arabidopsis thaliana* GN = Flowering-promoting factor 1 (FLP1) PE = 2 SV = 2	Swissprot	4.00×10^{-25}
Unigene0023748	Flowering-related B-class MADS (acronym referring to MCM1, AGAMOUS, DEFICIENS, SRF)-box protein [*Vitis vinifera*]	Nr	1.00×10^{-95}
Unigene0015337	K-box region and MADS-box transcription factor family protein [*Theobroma cacao*]	Nr	9.00×10^{-67}
Unigene0020944	K-box region and MADS-box transcription factor family protein isoform 1 [*Theobroma cacao*]	Nr	1.00×10^{-34}
Unigene0014037	MADS box transcription factor [*Elaeis guineensis*]	Nr	2.00×10^{-34}
Unigene0001006	MADS box transcription factor [*Populus tomentosa*]	Nr	9.00×10^{-74}
Unigene0014036	MADS1 [*Carica papaya*]	Nr	4.00×10^{-81}
Unigene0009074	MADS-box domain protein [*Camellia sinensis*]	Nr	2.00×10^{-52}
Unigene0026147	MADS-box protein 3 [*Vitis vinifera*]	Nr	2.00×10^{-38}
Unigene0007436	MADS-box protein AGL15 [*Dimocarpus longan*]	Nr	1.00×10^{-77}
Unigene0017569	MADS-box protein FLOWERING LOCUS C, Organism species (OS) = *Arabidopsis thaliana* GN = FLC PE = 2 SV = 1	Swissprot	1.00×10^{-38}
Unigene0041794	MADS-box protein SEP2B [*Aquilegia coerulea*]	Nr	9.00×10^{-26}
Unigene0026533	MADS-box protein suppressor of overexpression of constans 1 (SOC1), Organism species (OS) = *Arabidopsis thaliana* GN = SOC1 PE = 1 SV = 1	Swissprot	5.00×10^{-78}

Table S2. *Cont.*

Gene ID	Annotation	Annotation Databases	*E*-Value
Unigene0005810	MADS-box protein SVP OS = *Arabidopsis thaliana* GN = Short vegetative phase (SVP) PE = 1 SV = 1	Swissprot	3.00×10^{-73}
Unigene0021685	MADS-box transcription factor 16 OS = *Oryza sativa* subsp. *japonica*	Swissprot	2.00×10^{-15}
Unigene0001644	MADS-box transcription factor 27 OS = *Oryza sativa* subsp. *Japonica*	Swissprot	4.00×10^{-7}
Unigene0008775	Pedicel, carpel, stamen, petal differentiation and expansion stage, group 2-like protein [*Theobroma cacao*]	Nr	1.00×10^{-79}
Unigene0005239	Phytochrome and flowering time regulatory protein isoform 2 [*Theobroma cacao*]	Nr	1.00×10^{-24}
Unigene0041805	PREDICTED: MADS-box protein calmodulin binding 1 (CMB1) isoform 2 [*Vitis vinifera*]	Nr	4.00×10^{-55}
Unigene0016170	PREDICTED: MADS-box protein SVP [*Vitis vinifera*]	Nr	2.00×10^{-55}
Unigene0049385	Protein EARLY FLOWERING 3, Organism species (OS) = *Arabidopsis thaliana* GN = EARLY FLOWERING 3(ELF3) PE = 1 SV = 1	Swissprot	8.00×10^{-130}
Unigene0017817	Protein EARLY FLOWERING 4 OS = *Arabidopsis thaliana* GN = ELF4 PE = 1 SV = 1	Swissprot	1.00×10^{-27}
Unigene0003399	Sex determination protein tasselseed-2 OS = Zea mays GN = tasselseed-2 (TS2) PE = 2 SV = 1	Swissprot	8.00×10^{-59}
Unigene0021451	Uncharacterized protein involved in cell differentiation/sexual development	COG	2.00×10^{-24}
